# Editorial: Digital cognitive behavior therapy for obsessive-compulsive disorder

**DOI:** 10.3389/fnhum.2024.1396427

**Published:** 2024-03-28

**Authors:** Subho Chakrabarti

**Affiliations:** Post Graduate Institute of Medical Education and Research (PGIMER), Chandigarh, India

**Keywords:** digital, cognitive behavioral therapy, obsessive-compulsive disorder, videoconferencing, internet

Psychological treatments such as exposure and response prevention (ERP) or cognitive behavioral therapy (CBT) are effective in obsessive-compulsive disorder (OCD) either on their own or in combination with medications. However, barriers such as shortage of trained therapists, costs and other practical difficulties in obtaining treatment, unawareness, negative attitudes, and stigma impede access to conventional CBT or ERP. The rates of treatment-seeking are low, delays of several years are common, and a majority of the patients do not receive adequate in-person psychological treatment (Wootton, 2016). Digitally-based versions of these treatments have the potential to overcome the barriers to conventional psychotherapy by providing greater access for patients while saving costs and clinical resources (Castle et al., 2023). Effective forms of digital ERP or CBT have been developed over the past four decades (Aboujaoude, 2017). Currently, the two most common forms of these treatments are videoconferencing-based or internet-based CBT or ERP (Wootton, 2016; De Salazar et al., 2022). Both treatments appear to be equally effective. Videoconferencing-based treatments that are delivered live and synchronously resemble in-person treatments in terms of therapist contact, the structure of CBT or ERP, and the duration of sessions. They are more flexible and adaptable and provide greater access to the patient's home and family environments. Internet-based CBT (ICBT) has a broader and more robust evidence base supporting its efficacy and cost-effectiveness. Therapist or self-guided ICBT is more suited for wider dissemination of CBT for OCD. However, the amount of therapist contact which is an important determinant of outcome is quite variable. Therapist-guided ICBT provides sufficient contact resembling videoconferencing-based treatments, whereas self-guided ICBT has minimal or no therapist contact.

The four articles in this Research Topic provide insight into the different aspects of digital treatments for OCD. The bibliometric analysis by Tang et al. shows that research on OCD has steadily increased over the past two decades. One of the key areas is research on the treatment of OCD including CBT. However, the bulk of the research has been conducted in high-income countries. The authors propose that insufficient funding and inadequate quality of research may be reasons for the lack of contributions from low- and middle-income countries. The study of videoconferencing-based ERP by Murphy et al. is a good example of recent, large-scale naturalistic studies from clinical settings. An earlier study by the same group (Feusner et al., 2022) showed that videoconferencing-based ERP was effective and treatment gains were enduring. The treatment has a positive impact on comorbid depression and anxiety and the quality of life of the patients. It could reduce costs by reducing the therapist's time. However, high dropout rates are one of the main reasons for unsuccessful outcomes with digital psychotherapy (Aboujaoude, 2017). Therefore, the current study identified those at risk for non-engagement using a prediction algorithm and provided text-based peer support for this group. The patients who received this support spent more time in therapy and had greater reductions in symptom severity. The studies by this group show how the outcomes of digital treatments can be enhanced by innovative digital solutions for problems such as non-adherence that hinder the large-scale implementation of digital treatments for OCD. They also demonstrate the usefulness of hybrid care, which includes the blending of digital and in-person treatment as well as the use of multiple modes of digital technology in the treatment of OCD (Kayser et al., 2021; Feusner et al., 2022). A similar digital treatment using hybrid and enhanced care was examined by Wang et al. in their open trial of videoconferencing-based ERP in 25 children and adolescents. The treatment had a positive impact on comorbid symptoms, functional impairment, quality of life, and family accommodation. One of the principal problems affecting research on videoconferencing-based treatments for OCD is the lack of randomized controlled trials. Although open trials and naturalistic studies such as the ones included in this Research Topic show that videoconferencing-based ERP has wide-ranging benefits, reviews and meta-analyses of digital interventions for OCD include very few randomized trials of videoconferencing-based CBT or ERP (Wootton, 2016; De Salazar et al., 2022). The review of online group therapies for anxiety, OCD, and trauma-related disorders by Laurito et al. highlights this deficiency further. Although digitally-based group therapy was effective for social anxiety disorder and post-traumatic stress disorder, there were no studies of OCD as a primary condition.

The articles in this Research Topic suggest that digital CBT or ERP is a promising option for increasing the reach of effective psychological treatments for OCD. The evidence from observational studies and open trials of videoconferencing-based ERP indicates that enhanced and hybrid forms of digital treatments can be particularly useful in reducing the gap between those who need and those who receive adequate psychological treatments. However, the lack of properly conducted randomized trials suggests that the effectiveness of videoconferencing-based ERP is still unproven. Lastly, more research is needed from low- and middle-income countries where the treatment gap for OCD is larger and digital services are relatively underdeveloped.

## Author contributions

SC: Writing – original draft, Writing – review & editing.

## References

[B1] Aboujaoude E. (2017). Three decades of telemedicine in obsessive-compulsive disorder: a review across platforms. J. Obsessive. Compuls. Relat. Disord. 14, 65–70. 10.1016/j.jocrd.2017.06.003

[B2] Castle D. Feusner J. Laposa J. M. Richter P. M. A. Hossain R. Lusicic A.. (2023). Psychotherapies and digital interventions for OCD in adults: what do we know, what do we need still to explore? Compr. Psychiatry. 120:152357. 10.1016/j.comppsych.2022.152357 36410261 PMC10848818

[B3] De Salazar D. Pablo G. Pascual-Sánchez A. Panchal U. Clark B. Krebs G. (2022). Efficacy of remotely-delivered cognitive behavioural therapy for obsessive-compulsive disorder: an updated meta-analysis of randomised controlled trials. J. Affect. Disord. 322, 289–299. 10.1016/j.jad.2022.11.007 36395988

[B4] Feusner J. D. Farrell N. R. Kreyling J. McGrath P. B. Rhode A. Faneuff T.. (2022). Online video teletherapy treatment of obsessive-compulsive disorder using exposure and response prevention: clinical outcomes from a retrospective longitudinal observational study. J. Med. Internet Res. 24:e36431. 10.2196/36431 35587365 PMC9164091

[B5] Kayser R. R. Gershkovich M. Patel S. Simpson H. B. (2021). Integrating videoconferencing into treatment for obsessive-compulsive disorder: practical strategies with case examples. Psychiatr. Serv. 72, 840–844. 10.1176/appi.ps.202000558 33765864 PMC8249317

[B6] Wootton B. M. (2016). Remote cognitive-behavior therapy for obsessive-compulsive symptoms: a meta-analysis. Clin. Psychol. Rev. 43, 103–113. 10.1016/j.cpr.2015.10.001 26494179

